# The impact of virtual fracture clinics on medical education – a medical student perspective

**DOI:** 10.3402/meo.v21.30950

**Published:** 2016-03-15

**Authors:** Orlaith McAuliffe, Mariam Lami, Tamara Lami

**Affiliations:** 1 Faculty of Medicine, Imperial College London, South Kensington Campus, London, UK; 2 Faculty of Medicine, Imperial College London, London, UK; 3 Faculty of Life Sciences and Medicine, King's College London, London, UK

As year 5 medical students currently on a musculoskeletal placement, we have had the opportunity to observe orthopaedic consultants and nurses at work in ‘virtual fracture clinics’. This new service, recently launched, aims to review patients with potential fractures identified in acute care (1). Computers have enabled the remote assessment of these patients, reducing the need for out-of-hours fracture services and improving treatment times, saving both time and money for the National Health Service (NHS) (2–4). For patients, this service is particularly useful as their injuries are assessed much quicker; minor cases (where there is only a small fracture or no fracture identified) generally do not require a face-to-face consultation, and a telephone call with clear management instructions will normally suffice (5). This also means major cases are assessed and reviewed a lot quicker and more time can be spent on these patients in hospital fracture clinics. As a seemingly major advancement in patient care, we decided to delve deeper into the impact of these clinics, not on patients, but on medical students. Is there a place for virtual fracture clinics in medical education?

Firstly, virtual fracture clinics allow for more focused teaching. Students are exposed to a wider range of fractures in a shorter time period as cases are assessed much quicker than in normal clinics. This leaves time for the discussion of individual cases including the potential presentations and different management plans for fractures seen. Secondly, virtual clinics offer a patient-free learning space which allows students to query things they have seen more freely. This results in a more comfortable and interactive environment which is more engaging for both the doctor and the student. Practiced knowledge is shared in virtual fracture clinics and as we have experienced, there is much to be gleaned from being taught first-hand by an experienced specialist – an opportunity often missed in a clinic full of patients. Although valuable, there is only so much that we can learn from books; however, virtual clinics provide the ideal learning environment for practicing identifying fractures on radiographs – a notoriously difficult skill.

Medical education is vastly different between countries owing to the different structures of health care. However, medical schools worldwide are united in the use of problem-based learning (6). Virtual fracture clinics would allow medical students to be supervised by consultants in a clinical setting, integrating the teaching of both basic and clinical sciences – from the structure of bone to the management of the fracture or dislocation.

There has been a call for an international standardisation of medical education. Medical students across the globe have the right to learn, but the opportunities they receive are greatly influenced by the availability of staff and resources. Virtual fracture clinics allow for protected access to formal teaching worldwide, which may be particularly difficult in rural areas where the demand for specialists is high. In Japan and Australia for instance, this is certainly the case (6, 7). Orthopaedic services are often greatly concentrated in urban areas and teaching at rural hospitals is consequently neglected (7). Introducing virtual fracture clinics to rural areas and potentially developing countries would allow for remote specialist assessment of radiographs and thus students in these areas would still be able to reap the benefits from expert interpretations and management decisions.

On the contrary, diagnosing patients from a computer screen goes against everything we are taught at medical school. We are taught to treat ‘the patient’ not just ‘the fracture’, and in theory, virtual clinics may result in students overlooking the importance of building a doctor–patient rapport. As quoted by Plato, ‘the greatest mistake in the treatment of diseases is that there are physicians for the body and physicians for the soul, although the two cannot be separated’. In addition, the role of patient clinics in medical student education must also be acknowledged as the presentation of a fracture may not be the only concerning issue. The ability to take an accurate patient history is just as important as the ability to spot a fracture on an X-ray radiograph. Although beneficial for patients, the opportunity to send patient results for specialist review in virtual clinics may have a counterintuitive effect with medical students neglecting the need to learn to interpret radiographs themselves. This may be problematic as future doctors travelling abroad to work may not have the luxury of easily accessible specialists.

This all being said, the ability to recognise fractures is a highly specialised visual skill which benefits greatly from the intensified environment of a virtual clinic. Whilst holistic care might be a skill that is absent from these clinics, a balance can be struck. We would suggest that medical students be encouraged to attend both virtual fracture clinics and ordinary hospital fracture clinics to enhance their ability to diagnose fractures and to also ensure they do not lose out on building other vital clinical skills.

To all future medical students – ‘break a leg!’

*Orlaith McAuliffe* Faculty of Medicine Imperial College London South Kensington Campus London, UK Email: om511@ic.ac.uk *Mariam Lami* Faculty of Medicine Imperial College London London, UK *Tamara Lami* Faculty of Life Sciences and Medicine King's College London London, UK
